# Complete Genome Sequence of Salmonella enterica subsp. *diarizonae* Serovar 61:k:1,5,(7) Strain 14-SA00836-0, Isolated from Human Urine

**DOI:** 10.1128/MRA.00683-20

**Published:** 2020-09-03

**Authors:** Laura Uelze, Maria Borowiak, Antje Flieger, Sandra Simon, Simon H. Tausch, Burkhard Malorny

**Affiliations:** a German Federal Institute for Risk Assessment (BfR), Department for Biological Safety, Berlin, Germany; b Robert Koch Institute (RKI), Division of Enteropathogenic Bacteria and Legionella (FG11)/National Reference Centre for *Salmonella* and Other Bacterial Enteric Pathogens, Wernigerode, Germany; Indiana University, Bloomington

## Abstract

Salmonella enterica subsp. *diarizonae* serovar 61:k:1,5,(7) is commonly associated with sheep. Occasionally, the serovar has been found to also infect humans. Here, we report the complete genome sequence of strain 14-SA00836-0, isolated from human urine. To our knowledge, this is the first reported complete genome sequence of this serovar isolated from a human clinical sample.

## ANNOUNCEMENT

Salmonella enterica subsp. *diarizonae* serovar 61:k:1,5,(7) primarily infects sheep, with a high prevalence in sheep herds worldwide (1–3). Although most sheep appear to be asymptomatic carriers, infection with *Salmonella* serovar 61:k:1,5,(7) can cause chronic proliferative rhinitis (4, 5) and abortions (6, 7). The genome of a *Salmonella* serovar 61:k:1,5,(7) strain isolated from sheep was first described in 2019 (1). It was found to have a size of 4.88 Mbp and to contain one VirB4/D4 plasmid, as well as several prophage regions and novel genomic islands.

A small number of human cases involving severe infections have been described (8–10). Here, we report the complete genome sequence of a clinical *Salmonella* serovar 61:k:1,5,(7) isolate obtained in 2012. The strain was isolated from a urine sample from a 35-year-old female patient with urinary tract infection consulting a physician in Germany. The strain isolation was conducted by a diagnostic laboratory accredited according to DIN EN ISO 15189 and DIN EN ISO 17025. A pure culture of the strain was sent to the National Reference Centre for *Salmonella* and Other Bacterial Enteric Pathogens (NRC) at the Robert Koch Institute (RKI) for further serotyping. Sequencing was performed at the German Federal Institute for Risk Assessment (BfR).

The strain was cultivated for 18 h at 37°C on Endo agar, a selective medium for *Enterobacteria*, prior to purity control and further testing. Antimicrobial susceptibility testing was performed using broth microdilution following CLSI guidelines (CLSI M07-A9) and EUCAST epidemiological cutoff values (ECOFFs; https://mic.eucast.org/Eucast2/). The isolate was found to be susceptible to all tested antibiotics (ampicillin, azithromycin, cefotaxime, ceftazidime, chloramphenicol, ciprofloxacin, colistin, gentamicin, meropenem, nalidixic acid, sulfamethoxazole, tetracycline, tigecycline, and trimethoprim). Genomic DNA for short-read and long-read sequencing was isolated from an overnight liquid culture using the PureLink genomic DNA minikit (Invitrogen, Carlsbad, CA, USA). Sequencing libraries for Illumina short-read sequencing were prepared with the Nextera DNA Flex library prep kit and Nextera DNA CD indexes (Illumina, San Diego, CA, USA) according to the manufacturer’s protocol. Sequencing was performed in 2 × 151-bp cycles on the Illumina NextSeq benchtop sequencer using the NextSeq 500/550 midoutput kit v2.5 (300 cycles) (Illumina). A total number of 7,734,490 reads were generated, with 88.5% of bases above a quality score of 30 (Q > 30) and an overall coverage of 101.5×. The paired Illumina reads were trimmed using fastp v0.19.5 (11), yielding 7,534,722 reads.

To generate long reads for scaffolding, Oxford Nanopore MinION technology (Oxford, UK) was applied. MinION libraries were prepared using the rapid barcoding kit (Oxford Nanopore Technologies [ONT]), following the manufacturer’s instructions, and sequenced for approximately 16 h using a FLO-MIN106 R9 flow cell, generating 35,836 reads in total, with a read length *N*_50_ value of 10,434 bp and 56.0% of bases above a quality score of 10 (Q > 10). ONT Albacore Sequencing Pipeline Software v2.3.1 was used for base calling and quality control (QC) with default options.

The genome was hybrid assembled with Unicycler v0.4.4 (12), including Pilon v1.23 for polishing (13), providing the trimmed Illumina reads as paired short reads and the ONT reads as long reads with default parameters. Unicycler automatically identifies and trims overlaps for circular genomes and rotates the genome to begin with the *dnaA* gene. The 4.88-Mbp genome of *Salmonella* serovar 61:k:1,5,(7) isolate 14-SA00836-0 is composed of a circular chromosome of 4,832,352 bp (GC content, 51.5%) and a circular plasmid of 42,668 bp (GC content, 41.34%). A total of 4,535 coding DNA sequences (CDSs) and 4,330 coding genes were predicted within the chromosome using the NCBI Prokaryotic Genome Annotation Pipeline v4.11 (https://www.ncbi.nlm.nih.gov/genome/annotation_prok/) (14). No antimicrobial resistance genes were predicted with the BakCharak pipeline v1.0.0 (https://gitlab.com/bfr_bioinformatics/bakcharak), which implements ABRicate v1.0.1 (https://github.com/tseemann/abricate) and uses the NCBI resistance gene database.

### Data availability.

The genome assembly of isolate 14-SA00836-0 (SAMN15099378) was deposited in the NCBI GenBank database under the accession numbers CP054422.1 (chromosome) and CP054423.1 (plasmid). The sequencing reads are stored in the Sequence Read Archive under the accession numbers SRX8506662 (Nanopore) and SRX8506661 (Illumina). All data are encompassed under BioProject number PRJNA637259.
